# Tokyo 2020: A Sociodemographic and Psychosocial Characterization of the Portuguese Paralympic Team

**DOI:** 10.3390/healthcare10071185

**Published:** 2022-06-24

**Authors:** Tânia Mira, Diogo Monteiro, Aldo M. Costa, Pedro Morouço, Rui Matos, Raúl Antunes

**Affiliations:** 1 Department of Sport Sciences, University of Beira Interior, 6201-001 Covilhã, Portugal; taniaslmira@gmail.com (T.M.); amcosta@ubi.pt (A.M.C.); 2 ESECS, Polytechnic of Leiria, 2411-901 Leiria, Portugal; diogo.monteiro@ipleiria.pt (D.M.); pedro.morouco@ipleiria.pt (P.M.); rui.matos@ipleiria.pt (R.M.); 3 Research Center in Sport Sciences, Health Sciences and Human Development (CIDESD), 5001-801 Vila Real, Portugal; 4 CIEQV—Life Quality Research Centre, Polytechnic of Leiria, 2411-901 Leiria, Portugal; 5 Health Science Research Center (CICS-UBI), 6201-001 Covilhã, Portugal; 6 Center for Innovative Care and Health Technology (ciTechCare), Polytechnic of Leiria, 2411-901 Leiria, Portugal

**Keywords:** Paralympic Games, Tokyo 2020, sociodemographic, well-being, resilience, social support

## Abstract

The importance of practicing sports and its impact on the quality of life of people with disabilities is fundamental. Characterizing subjective well-being, resilience, and social influence in the practice of adapted sports, namely in those who participate in elite sport in Portugal, is truly important to support a set of initiatives to promote higher levels of practice. Thus, this study describes the Portuguese delegation at the Tokyo 2020 Paralympic Games through sociodemographic and psychosocial (positive and negative affect, life satisfaction, resilience, and social support) variables. The study involved 31 of the 33 athletes of the Portuguese Paralympic team aged between 15 and 58 years (34.45 ± 11.7 years), with 21 men and 10 women. Individual-level sociodemographic data gave us a clear insight into the reality of adapted sport in Portugal. The high values of life satisfaction, high positive affect and low negative affect, as well as high levels of resilience and social support seem to be important variables for these athletes. The data from the present study highlighted the importance of understanding the characteristics of Paralympic athletes, in order to better understand the reality of Paralympic sport in Portugal.

## 1. Introduction

One of the problems for people with disabilities is that from childhood they are not encouraged to have active lives, ending up living sedentary lives with significant health problems and barriers to physical activity [1]. Therefore, the physical inactivity of this population could increase the risk of developing secondary conditions, such as loneliness, fatigue, obesity [2]. A healthy lifestyle is as essential for promoting health and well-being and disease prevention for people without disabilities as for people with disabilities. Therefore, several authors [3,4] showed the importance of sports practice in people with disabilities.

In this regard, well-being has been one of the most studied variables. It gives people a better feeling of self-confidence, enthusiasm, leadership skills, and sociability. Happy people tend to be healthier, more efficient, successful, and they tend to volunteer in society [5,6].

Subjective well-being emerges as a subjective approach to quality of life [7,8]. People evaluate their life based on important domains (e.g., work, marriage, and health) or the affections and emotions they feel (e.g., joy, anxiety, and depression) [7,9,10,11,12]. Subjective well-being of the hedonic premise has a complex and multifaceted nature, and is divided into three components: satisfaction with life (cognitive), positive affect, and negative affect [10,13,14]. Cognitive assessments are characterized by life satisfaction and a sense of personal achievement, and affective appraisals assume the presence of positive affect (i.e., positive emotions and moods) and the absence of negative affect (i.e., negative emotions and moods) [6,7,15,16,17]. Positive affect is characterized by hedonic contentment experienced at a given moment, based on the description of an emotional state rather than on a cognitive judgment. In contrast, negative affect is characterized by a transitory state that includes negative experiences. In its turn, life satisfaction is a cognitive assessment that the person makes of certain areas of their life, depending on the comparison of real-life circumstances with what they define as a model [8,13].

Studies [18,19,20,21] revealed the positive associations between the practice of physical activity and increased well-being [12,22,23,24,25]. Some authors [26,27,28] showed the positive relationship between sports practice and subjective well-being in the disabled population. Well-being has been studied because of its role in actively coping with adversity [29]. Personal growth often involves experiences with obstacles, failure, and disappointment. Incidents such as these are necessary to find internal strengths and reintroduce resources while at the same time allowing one to become aware of one’s own limitations and vulnerabilities. This theme leads us to the study of resilience. In line with that, Fletcher and Sarkar [30] define resilience as “*The role of mental and behavioral processes in promoting personal assets and protecting the individual from the potential negative effect of stress*” (p. 675). Reacting positively to adversity depends on the hardships they have been subjected to and their respective adaptation [31]. Resilience is characterized as a dynamic process influenced by the environment and how the person relates to it, which allows for identifying the best attitude in each context [32].

With the increasing development and importance of the concept of resilience, many recent investigations have emerged within the scope of regular and adapted sports [30,33,34,35,36,37,38,39,40,41]. Participation in sports by people with disabilities has implications for resilience, access to social support, opportunities, and meaningful social experiences for people who faced traumatic injuries [42]. Athletes with disabilities show significant levels of resilience [42,43,44].

Due to inherent characteristics and problems, people with disabilities seem to be a vulnerable risk group for mental disorders such as depression, anxiety, stress, frustration, lack of motivation, and social impairment [45,46]. Many of the sports initiated during rehabilitation can be continued for pleasure throughout the life of the person with a disability. Pleasure is the primary motivational factor in the willingness to continue in sport [47]. People with physical disabilities who have participated in adapted sports have higher life satisfaction compared to people with physical disabilities who do not participate in any adapted sport [48,49,50]. People with disabilities who try to have an active lifestyle accept their disability better than inactive people; sport presents itself as a tool that promotes health, quality of life, social integration, self-confidence [48,49,51], satisfaction, quality of life, and self-esteem [48,49,51,52]. Sport decreases the suicidal tendencies of people with disabilities and promotes a more independent and motivated attitude [48,49].

Despite all the benefits mentioned, access to sports for people with disabilities is difficult due to the various barriers, which include a lack of understanding and awareness about inclusion, few opportunities and limited programs, inaccessibility of facilities, transportation difficulties, and lack of information and resources [51]. The support given to people with disabilities influences the promotion and maintenance of physical exercise in sports facilities [52]. Social support shows the person that they are loved, cared for, esteemed, and an integral part of a network of mutual obligations [53]. The origin of social support can be considered informal from family, friends, neighbors or social groups that accompany them daily, or formal when it comes from social institutions such as hospitals, doctors, social workers, and other specialists [54].

In studies that have been carried out on this topic, especially among young people, social support has been considered to be a positive influence in the sporting context [54]. Sheridan et al. [55] conducted a systematic review of social support in youth sports. They concluded that coaches, parents, and peers impact the development of youth sports through their positive influence on several factors. They also found that social support, over time, changed negatively, which can harm the athlete in both elite sport and physical activity [55]. This makes us aware of this need, properly in adjusting the athlete’s support pattern throughout the career.

More recently, Ascione et al. [56] referred to sport as a fundamental context in supporting the person with disabilities, contributing to enhancing and helping psychological issues that allow the development of their abilities. Through the practice of sports, the person with a disability and the others around them experience and assess their limits, using them positively as resources and qualities, accepting the difficulties.

In this regard, Martin [57] said that there are more studies with elite athletes, namely in adapted sports, since there is a significant difference between the number of studies published in regular sport than adapted sport. In addition, the variables under analysis in the present study (e.g., social support, well-being, and resilience) are important variables, particularly for this type of population, due to encouraging the participation and involvement of athletes using the strategies presented by Hellison [58]. This highlights the following: provide options for the activity to be performed (including the possibility of temporarily stopping the task/activity); allow the choice of the pace of participation, intensity and number of attempts; individualize and optimize the coach–athlete relationship through feedback, challenges and proposed activities. Moreover, Martin and Wheeler [59] stated that sport is an appropriate environment for these subjects to develop their own mechanisms in terms of resilience, since sometimes they are not accepted in other domains, and through sport they have the possibility to promote and develop their own skills, abilities, and personal resources, and consequently improve their coping strategies.

Thus, the present study aimed to characterize the Portuguese delegation at the Tokyo 2020 Paralympic Games through sociodemographic (age, gender, profession, education, and sports practice) and psychosocial variables (positive and negative affect, life satisfaction, resilience, and social support).

## 2. Materials and Methods

### 2.1. Study Design and Procedures

With the approval of the study by the ethics committee of the University of Beira Interior (CE-UBI-Pj-2018-076), there was initial contact with the Paralympic Committee of Portugal, to whom the study purpose was explained. Authorization was requested to carry it out with the Paralympic athletes who participated in the Tokyo 2020 Paralympic Games. Data were collected from questionnaires just after participation in the Tokyo 2020 Paralympic Games, between October and November 2021. The procedure explains the study’s objectives, and guarantees the principle of confidentiality. Informed consent was given by the participants, prior to data collection. Therefore, each athlete was provided with a link to access a Google Form, in which authorization was requested to carry out the study. The use of an online questionnaire was intended to facilitate the participation of all Portuguese Paralympic athletes, including athletes with visual impairments and athletes with intellectual disabilities, who in turn had the collaboration of coaches for this purpose. Regarding the completion of the questionnaires by the participants with intellectual disabilities (*n* = 4), the clubs were contacted in order to provide support in reading and understanding them, namely through specialists with training for the application of the questionnaires.

### 2.2. Participants

A total of 31 out of 33 athletes of the Portuguese Paralympic team aged between 15 and 58 years, with a mean age of 34.45 ± 11.7 years, with 21 men (36.29 ± 11.49 years) and 10 women (30.60 ± 11.84 years). Respondents were fully informed about the aim of the study. They were also told that they could stop at any time. Participants did not receive compensation for their participation.

### 2.3. Variables/Instruments

The sociodemographic questions were developed specifically for this study, having been reviewed by four experts. The other four questionnaires are validated instruments for the Portuguese population, evaluating four domains: sociodemographic data, life satisfaction, positive and negative affections, resilience, and social influence. To access information regarding the number of clubs in which there are sports modalities adapted for practice in Portugal, we accessed the website of the Portuguese Paralympic Committee, which contains a platform (Sport Inclusion Map) that allows us to find this information by searching by sport or geographical area [60].

To assess the Social Support perceived by athletes with disabilities, a scale based on the recommendations of Jago et al. [61], adapted with the objective assessment of children’s perceptions of friends and parental influences on physical activity, was divided into four dimensions: coach, parents, friends, and best friend. When asked about the support that the athlete has from their coach/parents/friends/best friend regarding exercise and sport (“encourages”, “practices”, “accompanies”, and “talks”), athletes responded on a Likert-type scale, with five levels, ranging from 1 (“rarely”) to 5 (“often”).

Subjective well-being was assessed through Satisfaction with Life Scale [62]. For the present study, the Portuguese version was used [63]. This scale presents five items (1. “In most ways my life is close to my ideal”, 2. “ The conditions of my life are excellent”, 3. “I am satisfied with my life”, 4. “ So far I have gotten the important things I want in life” and 5. “If I could live my life over again I would change almost nothing”), which are answered on a seven-point Likert scale, with 7 levels, ranging from 1 (“Strongly disagree”) to 7 (“Strongly agree”).

Positive and negative affect were evaluated through PANAS—The Positive and Negative Affect Schedule [64]. The Portuguese version of PANAS (PANAS-VRP) [65] was used in the present study. The PANAS-VRP presents 10 items (five items for positive affect: “inspired”, “alert”, “excited”, “enthusiastic” and “determined” and five items for negative affect: “fear”, “worried”, “nervous”, “scared” and “perturbed”) that are answered on a five-point Likert scale which varies between 1 (“Not at all or very slightly”) and 5 (“Extremely”).

To assess resilience, the Brief Resilience Scale (BRS) [66], in the Portuguese version [67], was used. This scale is composed of six items that are answered on a five-point Likert scale, as follows: (“1. I tend to recover quickly after difficult situations”, “2. I find it difficult to cope with stressful situations”, “3. I do not take much time to recover from a stressful situation”, “4. I find it difficult to recover quickly when something bad happens”, “5. I usually cope with difficult times without much worry” and “6. I tend to take a long time to overcome problems in my life”), with five levels, ranging from 1 (“I totally disagree”) to 5 (“I totally agree”).

### 2.4. Data Analysis

Considering the aim of the present study, a descriptive analysis was employed via SPPS v.27 (IBM, Armonk, NY, USA). Based on the central limit theorem, a sample greater than or equal to 30 approximates a normal distribution and therefore is often considered enough for the central limit theorem to hold [68]. In this regard, a descriptive analysis of location and central tendency measures (mean) and dispersion measures (standard deviation) were performed.

In addition, in order to measure the associations across studied variables, a Pearson bivariate correlation was performed. Cohen’s (1988) criterion was considered to interpret the magnitude of the correlation coefficients (r < 0.3 = low, r > 0.3 and <0.5 = medium, and r > 0.5 = high) [69] and the significance level to reject the null hypothesis was set at 5% [70].

## 3. Results

The sample’s sociodemographic characteristics that focus on the characterization of age, gender, disability, academic qualification, professional situation, type of sport, years of practice, weekly practice frequency, and training hours per week are presented in Table 1.

As can be seen in Table 1, the results are based on 31 athletes from eight Paralympic sports. Most athletes had motor disabilities.

The most common academic qualification is secondary education, followed by the third cycle of basic education. The first cycle is usually from between 6 and 9 years old, the second cycle is usually from between 10 and 12 years old and the third cycle is usually from between 13 and 15 years old.

Regarding the professional situation, the distribution is more varied. Most are employed or students and a small percentage are unemployed.

In relation to years of practice, it can be noted that about half of the sample has been practicing for more than 12 years. In relation to the number of training sessions per week the majority, they train more than five times per week and the number of hours of training per week is quite variable.

**Figure 1:**
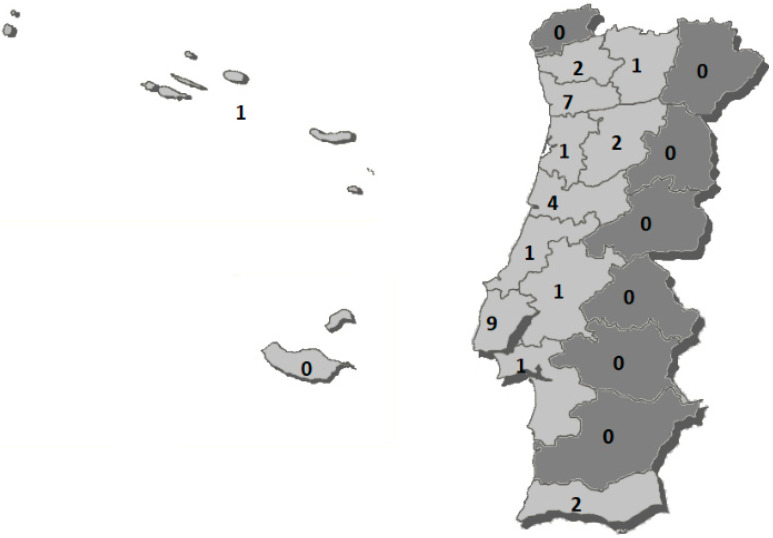
Distribution of athletes according to their districts in Portugal (light gray districts with athletes in the Tokyo 2020 Paralympic Games, dark gray districts without Paralympic athletes).

Figure 1 shows the distribution of athletes according to their residence districts. Thus, the majority of athletes reside in the districts of Lisbon and Porto.

**Figure 2:**
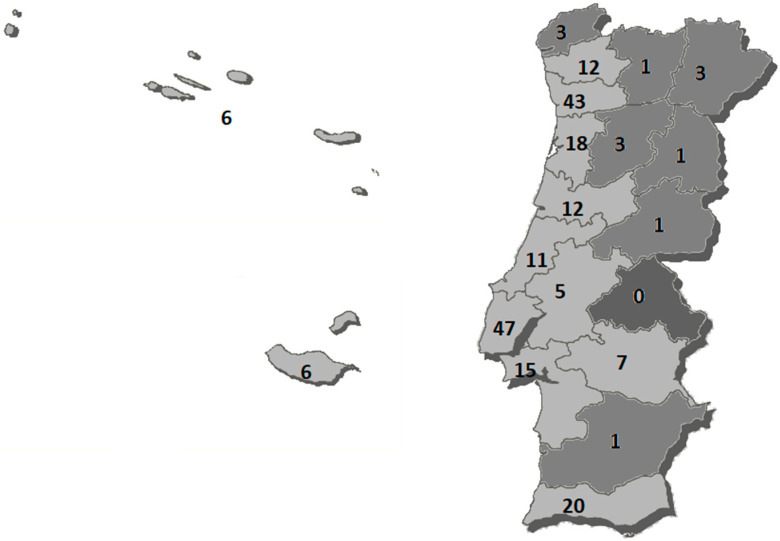
Distribution of adapted sports clubs by districts of Portugal.

On the Portuguese Paralympic Committee website platform, we found 204 clubs with sports adapted to practice in Portugal [60]. With this data, Lisbon and Porto are the districts with the most clubs (Figure 2). Still regarding the geographical distribution, it is worth noting the asymmetry between the coast and interior of Portugal, where most clubs are located in the coastal area of the country.

The variables of satisfaction with life, positive affect, negative affect, resilience, and social influence were analyzed and are presented in Table 2. Our results show that athletes perceive a positive affect superior to negative affect. Regarding social support, the perception of support by the coach is the one with the highest value.

Results for the association between different variables are summarized in Table 3.

The bivariate correlation was observed between life satisfaction and positive affect (medium), between positive affect with social support by the parents (high) and between positive affect with social support by the friends (medium). Resilience displayed a negative and significant association with the negative affect (high).

## 4. Discussion

The purpose of the present study was to describe the Portuguese delegation at the Tokyo 2020 Paralympic Games through sociodemographic (age, gender, disability, academic qualification, professional situation, type of sport, years of practice, weekly frequency, and training hours per week) and psychosocial variables (positive and negative affect, life satisfaction, resilience, and social support).

Thirty-three Portuguese athletes competing in eight sports participated in the 2020 Paralympics Games [71]. We found that 32.3% are female athletes and 67.7% are male athletes regarding sociodemographic data. This difference must, however, be analyzed considering that the number of women who practice sport or exercise regularly is lower than that of men [72]. On the other hand, there are no significant differences between the number of men and women with disabilities in the world [73].

While the 2020 Olympics were near gender parity for the first time, there are still deeply rooted gender stereotypes in Paralympic sport. Indeed, there has been a gradual increase in female participation in the Olympic Games, from 11% in Rome 1960 to around 45% in London 2012. However, in the Paralympic Games, it has increased much more slowly from a higher base of 21% in the Heidelberg 1972 Paralympics to 35% compared in London 2012 [74]. As early as 1995, Olenik et al. [75] noted that women with disabilities who aspire to reach the highest levels of sports performance face double discrimination—disability and gender discrimination.

When we analyze the weekly training hours of these athletes, the answers are pretty variable. Interestingly, the three athletes who train the most hours per week (more than 22 h) are in the same sport: swimming. Similarly, athletes who train the fewest hours per week (between 2 and 6 h) are boccia players. In a study conducted by Fagher et al. [76] with the Swedish Paralympic team, the average hours trained is 9 h per week.

As a suggestion for a future study, it would be interesting to analyze data on the number of weekly training hours of the other Paralympic teams for comparison.

Our results showed another important fact: only one athlete presents himself in the condition of unemployed. Few studies have been conducted on the elite athlete with a disability to the best of our knowledge. It is interesting how Bundon et al. [77] address this topic, noting that the research that has been done to understand how and why elite athletes with disabilities end their sporting careers. The research was conducted in the 1990s and is already outdated since the context has changed. There was concern about losing their income from the sport and there was little certainty about professional integration post-career in sport, mainly due to lack of work experience. These authors also found that the progression of certain types of disability may force some to leave the sport earlier than anticipated [77]. They discuss how previous generations of Paralympians struggled to find time to train while working or studying, paying the costs of their participation in sport. Currently, they have access to funding but face the difficulty of professional integration post-sports career [77,78,79]. The report of the Disability and Human Rights Observatory [80] showed a trend of growth of workers with disabilities in the public and private sectors.

We also noticed that seven districts do not present any athletes and, as we can see in the image in darker gray, they are primarily inland districts (Viana do Castelo, Bragança, Guarda, Castelo Branco, Portalegre, Évora and Beja). This may reflect the scarce supply and accessibility of adapted sports in the interior area at a national level. With the presented data, we can see that the districts that have more clubs with adapted sports disciplines are the districts with more athletes in the Paralympic Games: Porto and Lisbon. The islands are represented by the Azores with one athlete, and Madeira had no athletes participating in the Tokyo 2020 Games. Darcy [74] noted that resource-rich nations dominate medal-winning nations; he concluded that a more concerted effort must be made to help resource-poor governments strategically improve their participation rates in adapted sport. We can perceive our country’s needs at the level of the most resource-poor districts, be they structural or human. This is a robust practical implication of this work: knowing and identifying where the athletes are coming from and comparing this with the number of clubs is another contribution to the implementation of policies to promote adapted sports practice in our country. A competitive environment requires the elaboration of strategic actions that allow the achievement of pre-established objectives. It would be interesting to understand this reality by applying the model of Bosscher et al. [81] who designed the SPLISS (Sports Policies Leading to Sports Success) model, which compares and measures the effectiveness of national sports policies by training. They defined the critical factors that must be carried out for a country to increase the chances of international sports success. As we have seen, the most common academic qualification is secondary education, followed by the third cycle of primary education. Although the study conducted by the Directorate-General for Education and Science Statistics (DGEEC) [80] showed that in Portugal, the number of students with disabilities attending higher education increased by 57% compared with 2017/18, in our sample, the number of athletes with a university education is still far from the desired values. These results help us reflect on the need to encourage academic careers, making it possible to reconcile them with the dual career of sports training. The dual career aims to give the high-performance athlete the possibility to combine, in a fair way, a sporting career with an academic career [82].

Saphiro and Pitts [83], in their research, concluded that sports management scholars and practitioners do not identify sport, leisure, recreation and physical activity for the disabled as part of the business of the sport industry. Less than one-tenth of all published articles address sport for disability. There is a lack of information across the curriculum areas of sports management and in sport for people with disabilities. These authors suggest scholarship and advancement of studies in disability sport in sport business management.

It is important to increase the opportunities for disabled people through education, training, employment, transport, sports, and recreational activities. Therefore, investing in education centers in order to promote the practice of sports and its prolonged practice in these people are key issues not only in sports policy, but must be the position of sports in social policy in general [84].

The overall sample verified that athletes have high values of satisfaction with life, high positive affect, and low negative affect. These results seem to align with previous studies, which concluded that Paralympic athletes have interactions with others, disabled and non-disabled athletes, that give them opportunities to establish new relationships and friendships, increasing life satisfaction [85].

These results for subjective well-being are interesting; studies point to the fact that affect and life satisfaction allow for a significant increase in health, longevity, work, earnings, social relationships, and benefits to society [6]. Côté-Leclerc et al. [86], in their study, found that the positive effect of adapted sport on the quality of their lives acts mainly through personal factors (behavior and health), social participation (interpersonal relationships) and the environment (societal perception and support of the environment). Well-being gives people higher self-confidence, enthusiasm, leadership skills and sociability. Happy people tend to be healthier, more efficient, successful and they tend to volunteer more in society, businesses, and other organizations, individual and governmental, enabling them to increase performance [6,7]. Similar results were found by Hammond’s study [87] which analyzed these variables in Paralympic athletes and concluded that they represent a very high functioning group within the population, with high levels of subjective wellbeing. In other study, Silva et al. [88] showed that the subjective well-being are positively affected by sport participation of athletes with disabilities.

We also found a high value for resilience. This data reinforces the literature that states athletes with disabilities show significant levels of resilience [42,43,44,86]. Hariharan et al. [89] found that resilient people with disabilities had higher emotional intelligence and more positive perceptions of their environment. These positive perceptions of emotional resources allow people with disabilities to overcome barriers and difficulties. In their experiences, athletes face countless hours of training, often repetitive and with implications for stress levels, time to recover from injuries that prevent them from performing, and competitive anxiety with the agony of failure. For these reasons, athletes need physical stamina, talent, and mental toughness [21,90,91]. Thus, they must possess a greater ability to cope with challenges and adversity.

In what concerns social support for physical activity or sport’s practice, we verified that the coach has the highest value of the most influence on the athlete, followed by friends, best friends and, lastly, parents. In recent years, studies on social support have increased considerably; from family, friends, and coaches [92,93] to a variety of support staff in a multidisciplinary team [94,95], social support is essential for well-being, allows better integration in society and better achievement of goals. Banack et al. [96], reinforce the importance of the relationship of the Paralympic athlete with the coach in the support of autonomy. Many of the athletes, depending on the disability, need the coach as an assistant in basic activities in training and in competition. Although in our study parental support appears last in degree of importance, Shapiro and Malone [97] refer to family support as extremely important, especially in younger athletes. This result may have to do with the fact that the athletes are older than 16. Social support relationships are all important for access to sport by people with disabilities however, the higher the competitive level, the greater the social support relationships [98].

Our results showed that there is an association between life satisfaction and positive affect, which is in line with the conceptual framework of subjective well-being [7,11,13,62] and with some studies conducted recently [99,100]. The association between the positive effect and the social support of parents and friends reinforces the importance that this support seems to have in the emotional states of the athletes, confirming some of the evidence in the literature [97,98].

Regarding resilience and its negative association with negative affect seems to indicate a possible buffer effect of resilience for negative emotional experiences [101,102,103]. Although these associations are interesting, they need to be clarified in future studies.

Despite the importance of the present study, some limitations must be acknowledged and should be addressed in future studies. This study was conducted in Portugal, thus results cannot be generalized to other countries and contexts. It will also be very interesting to relate, in future studies, the psychological variables (e.g., well-being, resilience, social support) with demographic variables and with the variables of sports practice (e.g., years of practice). However, this work may have an important contribution to understanding the reality of Paralympic sport in Portugal.

## 5. Conclusions

The sociodemographic (age, gender, profession, education, sports practice) and psychosocial (positive and negative affect, life satisfaction, resilience, and social support) variables could characterize the Tokyo 2020 Paralympic team. The disparity between the team’s total number of men and women is still a reality. Training hours per week are pretty heterogeneous among athletes. The offer and accessibility to adapted sports at the national level are considerably lower in the inner part of the country.

In the sample, we found that Portuguese Paralympic athletes have high values of life satisfaction, high positive affect, low negative affect, and good levels of resilience. Additionally, our results showed that the coach has the most decisive influence on the athlete, followed by friends, best friends, and parents. The coach is indeed the most critical figure in this social influence.

These findings are important and could be considered for further analysis and evaluation of the reality of adapted sport in Portugal, supporting the idea that more public development policies are needed for people with disabilities to access adapted physical activity and sport.

## Figures and Tables

**Table 1:** Summary of the descriptive statistics for the sample’s sociodemographic characteristics (*n* = 31).

Variables	*n* (%)	Mean ± SD
	31	
**Age (Years)**		34.45 ± 11.7
**Gender**		
Male	21 (67.7%)	
Female	10 (32.3%)	
**Disability**		
Motor	24 (77.4%)	
Visual	3 (9.7%)	
Intellectual	4 (12.9%)	
**Academic qualification**		
1st Cycle of Basic Education	1 (3.2%)	
2nd Cycle of Basic Education	1 (3.2%)	
3rd Cycle of Basic Education	5 (16.1%)	
Upper Secondary Education	17 (54.8%)	
Bachelor’s degree	4 (12.9%)	
Undergraduate degree	1 (3.2%)	
Master’s degree	2 (6.5%)	
Ph.D. degree	0 (0%)	
**Professional situation**		
Student	9 (29%)	
Public service	5 (16.1%)	
Outsourced account	4 (12.9%)	
Personal account	3 (9.7%)	
Unemployed	1 (3.2%)	
Retired	3 (9.7%)	
Other	6 (19.4%)	
**Type of Sport**		
Para Athletics	9 (29%)	
Para Badminton	1 (3.2%)	
Boccia	9 (29%)	
Para Canoe	2 (6.5%)	
Para Cycling	2 (6.5%)	
Equestrian	1 (3.2%)	
Judo	1 (3.2%)	
Para Swimming	6 (19.4%)	
**Years of practice**		
4 to 7 years	4 (12.9%)	
8 to 11 years	11 (35.5%)	
12 or more	16 (51.6%)	
**Weekly training frequency**		
3 per week	4 (12.9%)	
4 per week	3 (9.7%)	
5 per week	3 (9.7%)	
More than 5 per week	21 (67.7%)	
**Training hours per week**		
2 to 6 h	6 (19.4%)	
7 to 10 h	3 (9.7%)	
11 to 14 h	8 (25.8%)	
15 to 18 h	7 (22.6%)	
19 to 22 h	4 (12.4%)	
More than 22 h	3 (9.7%)	

Note: SD, standard deviation.

**Table 2:** Summary of the descriptive statistics for the sample variables (*n* = 31).

Variables	Mean		
	Mean ± SD	(95% CI)	Median (IQR)
**Life Satisfaction**	5.24 ± 0.97	4.88–5.59	5.00 (1.00)
**Positive affect**	3.96 ± 0.64	3.73–4.20	4.00 (0.60)
**Negative affect**	1.72 ± 0.64	1.48–1.95	1.60 (1.00)
**Resilience**	3.73 ± 0.77	3.45–4.01	3.80 (1.00)
**Social Support**			
Coach	3.48 ± 0.58	3.26–3.69	3.50 (0.75)
Parents	2.55 ± 0.89	2.22–2.88	2.50 (1.25)
Friends	3.00 ± 0.76	2.72–3.28	3.00 (1.00)
Best Friend	2.83 ± 0.94	2.49–3.18	3.00 (1.75)

Notes: SD, standard deviation; 95% CI, confidence interval 95%; IQR, interquartile range.

**Table 3:** Bivariate correlations between variables.

	1	2	3	4	5	6	7	8
**1. Life Satisfaction**	1	-	-	-	-	-	-	-
**2. Positive affect**	0.47 **	1	-	-	-	-	-	-
**3. Negative affect**	−0.18	−0.05	1	-	-	-	-	-
**4. Resilience**	0.28	0.08	−0.53 **	1	-	-	-	-
**5. Social Support—Coach**	0.08	0.37	0.06	0.01	1	-	-	-
**6. Social Support—Parents**	0.13	0.51 **	−0.19	0.18	0.34	1	-	-
**7. Social Support—Friends**	0.26	0.43 *	0.12	0.22	0.34	0.27	1	-
**8. Social Support—Best Friend**	0.06	0.26	−0.15	0.12	0.30	0.23	0.62 **	1

**. *p* < 0.001; *. *p* < 0.05.

## Data Availability

Additional data are available upon request to the author for correspondence.

## References

[1] Saphiro D., Martin J. (2010). Multidimensional Physical Self-Concept of Athletes with Physical Disabilities. Adapt. Phys. Act. Q..

[2] Laskowski E. (2012). The role of exercise in the treatment of obesity. PMR.

[3] Slater D., Meade M.A. (2004). Participation in recreation and sports for persons with spinal cord injury: Review and recommendations. NeuroRehabilitation.

[4] Medola F., Busto R., Marçal A., Júnior A., Dourado A. (2011). Sports on quality of life of individuals with spinal cord injury: A case series. Rev. Bras. Med. Esporte.

[5] Diener E., Kesebir P., Lucas R. (2000). Benefits of accounts of well-being—For societies and for psychological science. Appl. Psychol. Int. Rev..

[6] Ryan R.M., Deci E.L. (2001). On happiness and human potentials: A review of research on hedonic and eudaimonic well-being. Annu. Rev. Psychol..

[7] Diener E. (2000). Subjective well-being—The science of happiness and a proposal for a national index. Am. Psychol..

[8] Albuquerque A., Tróccoli B. (2004). Desenvolvimento de uma escala de bem-estar subjetivo. Psicol. Teor. Pesqui..

[9] Diener E. (1994). Assenssing subjective well-being—Progress and opportunities. Soc. Indic. Res..

[10] Diener E., Suh E.M., Lucas R.E., Smith H.L. (1999). Subjective well-being: Three decades of progress. Psychol. Bull..

[11] Diener E., Ryan K. (2009). Subjective well-being: A general overview. S. Afr. J. Psychol..

[12] Moraes M., Corte-Real N., Dias C., Fonseca A.M. (2012). Um olhar sobre a prática desportiva, bem-estar subjetivo e integração social de imigrantes… em Portugal e no mundo. Psicol. Soc..

[13] Diener E., Oishi S., Lucas R.E. (2003). Personality, culture, and subjective well-being: Emotional and cognitive evaluations of life. Annu. Rev. Psychol..

[14] Ryff C., Keyes C. (1995). The Structure of Psychological Well-Being Revisited. J. Personal. Soc. Psychol..

[15] Dias C., Corte-Real N., Corredeira R., Barreiros A., Bastos T., Fonseca A. (2008). A prática desportiva dos estudantes universitários e suas relações com as autopercepções físicas, bem-estar subjectivo e felicidade. Estud. Psicol..

[16] Giacomoni C. (2004). Bem-estar subjetivo: Em busca da qualidade de vida. Temas Psicol. SBP.

[17] Waterman A.S. (1993). Two conceptions of happiness: Contrasts of personal expressiveness (eudaimonia) and hedonic enjoyment. J. Personal. Soc. Psychol..

[18] Caddick N., Smith B. (2014). The impact of sport and physical activity on the well-being of combat veterans: A systematic review. Psychol. Sport Exerc..

[19] Hogan C.L., Catalino L.I., Mata J., Fredrickson B.L. (2015). Beyond emotional benefits: Physical activity and sedentary behaviour affect psychosocial resources through emotions. Psychol. Health.

[20] Mack D.E., Wilson P.M., Gunnell K.E., Gilchrist J.D., Kowalski K.C., Crocker P.R.E. (2012). Health-Enhancing Physical Activity: Associations with Markers of Well-Being. Appl. Psychol. Health Well Being.

[21] Smith A.L., Ntoumanis N., Duda J.L., Vansteenkiste M. (2011). Goal Striving, Coping, and Well-Being: A Prospective Investigation of the Self-Concordance Model in Sport. J. Sport Exerc. Psychol..

[22] Downward P., Rasciute S. (2011). Does sport make you happy? Na analysis of the well-being derived from sports participation. Int. Rev. Appl. Econ..

[23] Ku P., Kenneth F., Chang C., Sun W., Chen L. (2014). Cross-Sectional and Longitudinal Associations of Categories of Physical Activities with Dimensions of Subjective Well-being in Taiwanese Older Adults. Soc. Indic. Res..

[24] Ku P., McKenna J., Fox K.R. (2007). Dimensions of Subjective Well-being and Effects of Physical Activity in Chinese Older Adults. J. Aging Phys. Act..

[25] Olsson L., Hurtig-Wennlof A., Nilsson T. (2014). Subjective well-being in Swedish active seniors and its relationship with physical activity and commonly available biomarkers. Clin. Interv. Aging.

[26] Albrecht G., Devlieger P. (1999). The disability paradox: High quality of life against all odds. Soc. Sci. Med..

[27] Chow S., Lo S.K., Cummins R. (2005). Self-perceived quality of life of children and adolescents with physical disabilities in Hong Kong. Qual. Life Res..

[28] Emerson E., Honey A., Madden R., Llewellyn G. (2009). The Well-Being of Australian Adolescents and Young Adults with Self-Reported Long-Term Health Conditions, Impairments or Disabilities: 2001 and 2006. Aust. J. Soc. Issues.

[29] Ryff C.D. (2014). Self-realisation and meaning making in the face of adversity: A eudaimonic approach to human resilience. J. Psychol. Afr..

[30] Fletcher D., Sarkar M. (2012). A grounded theory of psychological resilience in Olympic Champions. Psychol. Sport Exerc..

[31] Morgan P.B.C., Fletcher D., Sarkar M. (2013). Defining and characterizing team resilience in elite sport. Psychol. Sport Exerc..

[32] Angst R. (2009). Psicologia e resiliência: Uma revisão da literatura. Psicol. Argum..

[33] Bejan R., Tonita F. (2013). The role of the resilience in coping with stress in sports. Soc. Behav. Sci..

[34] Besharat M.A. (2010). Relationship of Alexithymia with Coping Styles and Interpersonal Problems. Procedia-Soc. Behav. Sci..

[35] Cevada T., Cerqueira L.S., de Moraes H.S., dos Santos T.M., Pompeu F., Deslandes A.C. (2012). Relationship between sport, resilience, quality of life, and anxiety. Rev. Psiquiatr. Clin..

[36] Fontes R.D.D., Brandao M.R.F. (2013). Resilience in sport: An ecological perspective on human development. Mot. Rev. Educ. Fis..

[37] Fletcher D., Sarkar M. (2013). Psychological Resilience A Review and Critique of Definitions, Concepts, and Theory. Eur. Psychol..

[38] Lu F.J.H., Lee W.P., Chang Y.K., Chou C.C., Hsu Y.W., Lin J.H., Gill D.L. (2016). Interaction of athletes’ resilience and coaches’ social support on the stress-burnout relationship: A conjunctive moderation perspective. Psychol. Sport Exerc..

[39] Neves A., Hirata K., Tavares M. (2015). Imagem corporal, trauma e resiliência: Reflexões sobre o papel do professor de Educação Física. Rev. Quadrimestral Assoc. Bras. Psicol. Esc. Educ..

[40] Nicholls A., Morley D., Perry J. (2016). The Model of Motivational Dynamics in Sport: Resistance to Peer Influence, Behavioral Engagement and Disaffection, Dispositional Coping, and Resilience. Front. Psychol..

[41] Sarkar M., Fletcher D. (2014). Psychological resilience in sport performers: A review of stressors and protective factors. J. Sports Sci..

[42] Machida M., Irwin B., Feltz D. (2013). Resilience in Competitive Athletes With Spinal Cord Injury: The Role of Sport Participation. Qual. Health Res..

[43] Cardoso F.L., Sacomori C. (2013). Resilience of athletes with physical disabilities: A cross-sectional study. Rev. Psicol. Deporte.

[44] Sirorska I., Gerc K. (2018). Athletes with disability in the light of positive psychology. Balt. J. Health Phys. Act..

[45] Ferreira J., Fox K. (2008). Physical self-perceptions and self-esteem in male basketball players with and without disability: A preliminar analysis using the physical self-perception profile. Eur. J. Adapt. Phys. Act..

[46] Sahlin B., Lexell J. (2015). Impact of Organized Sports on Activity, Participation, and Quality of Life in People with Neurologic Disabilities. PMR.

[47] Martin J.J. (1996). Transitions out of competitive sport for athletes with disabilities. Ther. Recreat. J..

[48] Frank C., Land W.M., Schack T. (2013). Mental representation and learning: The influence of practice on the development of mental representation structure in complex action. Psychol. Sport Exerc..

[49] Blauwet C., Willick S.E. (2012). The Paralympic Movement: Using Sports to Promote Health, Disability Rights, and Social Integration for Athletes with Disabilities. PMR J. Inj. Funct. Rehabil..

[50] Yazicioglu K., Yavuz F., Goktepe A., Tan A. (2012). Influence of adapted sports on quality of life and life satisfaction in sport participants and non-sport participants with physical disabilities. Disabil. Health J..

[51] Misener L., Darcy S. (2014). Managing disability sport: From athletes with disabilities to inclusive organisational perspectives. Sport Manag. Rev..

[52] Javorina D., Shirazipour A., Allan V., Latimeur-Cheung A. (2020). The impact of social relationships on initiation in adapted physical activity for individuals with acquired disabilities. Psychol. Sport Exerc..

[53] Cobb S. (1976). Social support as a moderator of life stress. Psychosom. Med..

[54] Dunst C., Trivette C., Meisels S., Shonkoff J. (1990). Assessment of social support in early intervention programs. Handbook of Early Childhood Intervention.

[55] Sheridan D., Coffee P., Lavallee D. (2014). A systematic review of social support in youth sport. Int. Rev. Sport Exerc. Psychol..

[56] Ascione A., Belfiore P., Di Palma D. (2018). Sports program to promote the well-being of people with disabilities. Acta Med. Mediterr..

[57] Martin J. (2018). Handbook of Disability Sport and Exercise Psychology.

[58] Hellison D. (1995). Teaching Responsability through Physical Activity.

[59] Martin J., Wheeler G., Vanlandewijck Y., Thompson W. (2011). Psychology. Handbook of Sports Medicine and Science—The Paralympic Athlete.

[60] Comité Olímpico de Portugal. https://paralimpicos.pt/mapa-inclusao-desportiva.

[61] Jago R., Fox K., Page A., Brockman R., Thompson J. (2009). Development of scales to assess children’s perceptions of friend and parental influences on physical activity. Int. J. Behav. Nutr. Phys. Act..

[62] Diener E., Emmons R.A., Larsen R.J., Griffin S. (1985). The Satisfaction with Life Scale. J. Personal. Assess..

[63] Neto F. (1993). The satisfaction with life scale: Psychometrics properties in an adolescent sample. J. Youth Adolesc..

[64] Watson D., Clark L.A., Tellegen A. (1988). Development and validation of brief measures of positive and negative affect: The PANAS scales. J. Personal. Soc. Psychol..

[65] Galinha I., Pereira C., Esteves F. (2014). Versão reduzida da escala portuguesa de afeto positivo e negative—PANAS-VRP: Análise fatorial confirmatória e invariância temporal. Rev. Psicol..

[66] Smith B.W., Dalen J., Wiggins K., Tooley E., Christopher P., Bernard J. (2008). The brief resilience scale: Assessing the ability to bounce back. Int. J. Behav. Med..

[67] Silva-Sauer L., Torre-Luque A., Smith B.W., Lins M.C., Andrade S., Fernández-Calvo B. (2020). Brief resilience scale (brs) portuguese version: Validity and metrics for the older adult population. Aging Ment. Health.

[68] Hair J.F., Black W.C., Babin B.J., Anderson R.E. (2019). Multivariate Data Analysis. www.cengage.com/highered.

[69] Cohen J. (1988). Statistical Power Analysis for the Behavioral Sciences.

[70] Ho R. (2014). Handbook of Univariate and Multivariate Data Analysis with IBM SPSS.

[71] Comité Paralímpico de Portugal. https://comiteolimpicoportugal.pt/wp-content/uploads/Toquio-2020-Guia-Desportivo.pdf.

[72] World Health Organization & World Bank (2011). World Report on Disability 2011.

[73] European Opinion Research Group (2018). Special Eurobarometer 472.

[74] Darcy S., Brittain I., Beacom A. (2018). “Behemoths and the Also-Rans”: The International Paralympic Movement as a Pyramid Built on Quicksand. The Palgrave Handbook of Paralympic Studies.

[75] Olenik L., Matthews J., Steadward R. (1995). Women, Disability and Sport: Unheard Voices. Canadian Woman Studies.

[76] Fagher K., Dahstrom O., Jacobsson J., Timpka T., Lexell J. (2020). Injuries and illnesses in Swedish Paralympic athletes—A 52-week prospective study of incidence and risk factors. Scand. J. Med. Sci. Sports.

[77] Bundon A., Ashfield A., Smith B., Goosey-Tolfrey V. (2018). Struggling to stay and struggling to leave: The experiences of elite para-athletes at the end of their sport careers. Psychol. Sport Exerc..

[78] Wheeler G.D., Malone L.A., VanVlack S., Nelson E.R., Steadward R.D. (1996). Retirement from disability sport: A pilot study. Adapt. Phys. Act. Q..

[79] Wheeler G.D., Steadward R.D., Legg D., Hutzler Y., Campbell E., Johnson A. (1999). Personal investment in disability sport careers: An international study. Adapt. Phys. Act. Q..

[80] Observatório da Deficiência e direitos Humanos (2021). Pessoas com Deficiência em Portugal.

[81] De Bosscher V., De Knop P., Van Bottenburg M., Shibli S. (2006). A Conceptual Framework for Analysing Sports Policy Factors Leading to International Sporting Success. Eur. Sport Manag. Q..

[82] Picamilho S., Saragoça J., Teixeira M. (2021). Dual careers in high sporting performance in europe: A systematic literature review. Motricidade.

[83] Saphiro D., Pitts B. (2014). What Little Do We Know: Content Analysis of Disability Sport in Sport Management Literature. J. Sport Manag..

[84] Walker C.M., Hayton J.W. (2017). Navigating austerity: Balancing ‘desirability with viability’in a third sector disability sports organisation. Eur. Sport Manag. Q..

[85] Ondrušova Z., Pitekova R., Bardiovsky M., Galikova Z. (2013). Sport and doing sport s by the disabled posttraumatic return to life. Clin. Soc. Work.

[86] Côté-Leclerc F., Duchesne G., Bolduc P., Gélinas-Lafrenière A., Santerre C., Desrosiers J., Levasseur M. (2017). How does playing adapted sports affect quality of life of people with mobility limitations? Results from a mixed-method sequential explanatory study. Health Qual. Life Outcomes.

[87] Hammond T. (2014). The Subjective Well-Being of Paralympic Athletes.

[88] Silva A., Monteiro D., Sobreiro P. (2021). Effects of sports participation and the perceived value of elite sport on subjective well-being. Sport Soc..

[89] Hariharan M., Karimi M., Kishore M.T. (2014). Resilience in persons with disabilities: Role of perceived environment and emotional intelligence. J. Indian Acad. Appl. Psychol..

[90] Jones G., Hanton S., Connaughton D. (2002). What Is This Thing Called Mental Toughness? An Investigation of Elite Sport Performers. J. Appl. Sport Psychol..

[91] Vallerand R.J., Losier G.F. (1999). An integrative analysis of intrinsic and extrinsic motivation in sport. J. Appl. Sport Psychol..

[92] Rosenfeld L., Richman J., Hardy C. (1989). Examining social support networks among athletes: Description and relationship to stress. Sport Psychol..

[93] Barefield S., McCallister S. (1997). Social support in the athletic training room: Athletes’ expectations of staff and student athletic trainers. J. Athl. Train..

[94] Gould D., Guinan D., Greenleaf C. (1999). Factors affecting Olympic performance: Perceptions of athletes and coaches from more and less successful teams. Sport Psychol..

[95] Burns L., Weissensteiner J., Cohen M. (2019). Lifestyles and mindsets of Olympic, Paralympic and world champions: Is an integrated approach the key to elite performance?. Br. J. Sports Med..

[96] Banack H., Sabiston C., Bloom G. (2011). Coach Autonomy Support, Basic Need Satisfaction, and Intrinsic Motivation of Paralympic Athletes. Res. Q. Exerc. Sport.

[97] Shapiro D., Malone L. (2016). Quality of life and psychological affect related to sport participation in children and youth athletes with physical disabilities: A parent and athlete perspective. Disabil Health J..

[98] VaezMousavia M., Mousavib A., Mohammadic F. (2021). Psychological Characteristics of Iranian Para-athletes. Int. J. Mot. Control Learn..

[99] Busseri M.A. (2018). Examining the structure of subjective well-being through meta-analysis of the associations among positive affect, negative affect, and life satisfaction. Personal. Individ. Differ..

[100] Jovanović V., Joshanloo M. (2021). The Contribution of Positive and Negative Affect to Life Satisfaction across Age. Appl. Res. Qual. Life.

[101] Ryff C.D., Singer B., Keyes C.L.M., Haidt J. (2003). Flourishing under fire: Resilience as a prototype of challenged thriving. Flourishing: Positive Psychology and the Life Well-Lived.

[102] Ong A.D., Bergeman C.S., Bisconti T.L., Wallace K.A. (2006). Psychological resilience, positive emotions, and successful adaptation to stress in later life. J. Personal. Soc. Psychol..

[103] Cohn M.A., Fredrickson B.L., Brown S.L., Mikels J.A., Conway A.M. (2009). Happiness unpacked: Positive emotions increase life satisfaction by building resilience. Emotion.

